# Supplementary figures and images for: Liver slice culture as a model for lipid metabolism in fish
Source: PeerJ. 2019 Sep 17;7:e7732. doi: 10.7717/peerj.7732 (PMC6753922; doi:10.7717/peerj.7732)

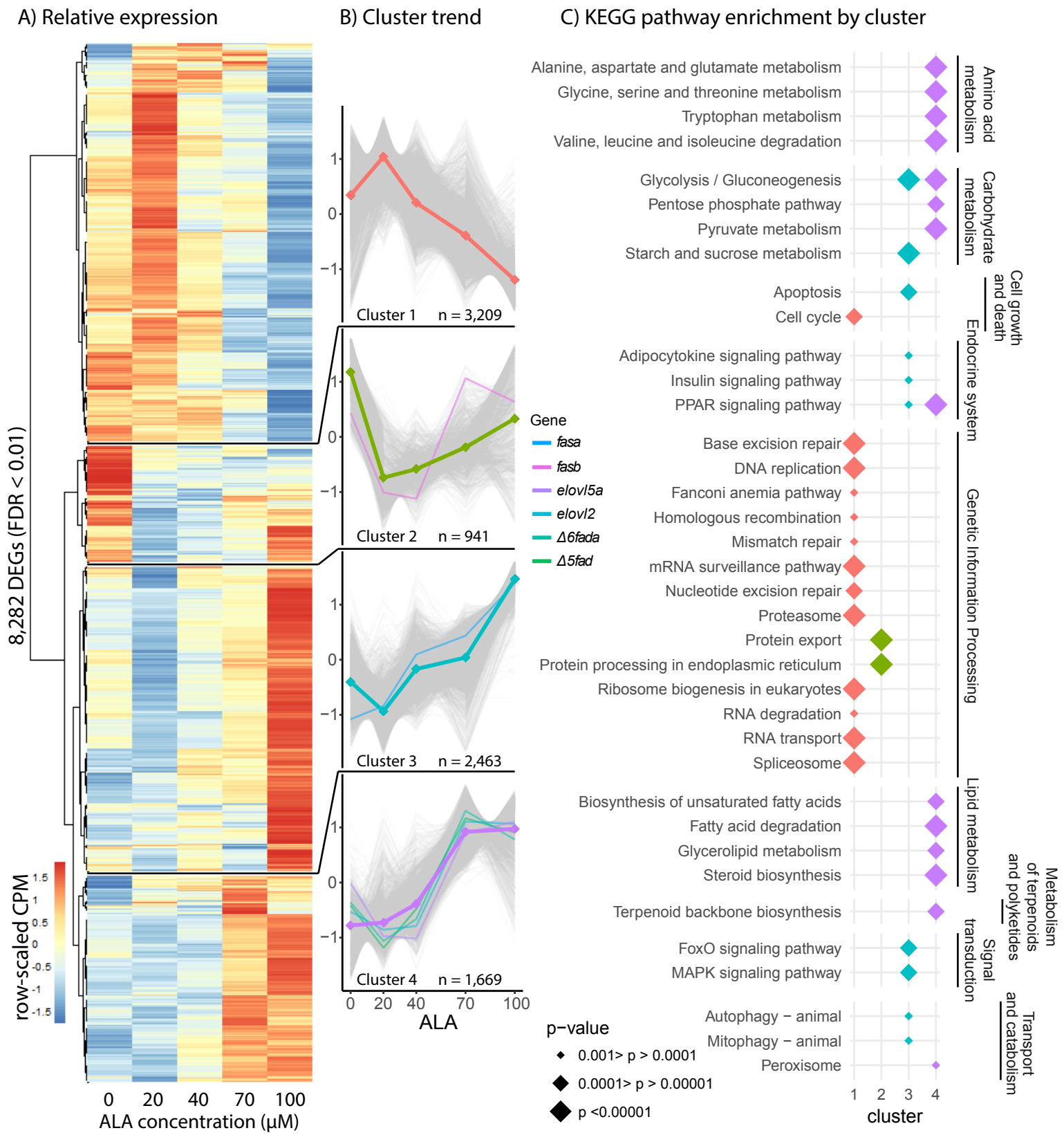

Supplement: Supplemental Information 1 — (A) Heatmap showing changes in relative gene expression with increasing concentration of ALA. Heatmap contains 8,282 genes differentially expressed (FDR < 0.01) at any point in the ALA gradient (ANOVA-like). Transcript abundance is expressed CPM and were row-scaled to highlight changes in individual gene expression. Genes were broken up into four clusters with similar expression patterns across the ALA gradient. (B) Mean row-scaled CPM (thick lines with points) colored by cluster overlays row-scaled CPM of individual genes (gray). Expression of fatty acid synthase a (fasa), fatty acid synthase b (fasb), elongase 5a (elovl5a), elongase 2 (elovl2), Δ 6 desaturase a (Δ6fada), and Δ 5 desaturase (Δ5fad) are also plotted (thin lines colored by gene). (C) Pathway enrichment analysis on each gene cluster. All pathways shown are significantly enriched (p < 0.001) in the corresponding expression cluster with point size corresponding to p-value. [file peerj-07-7732-s001.pdf]

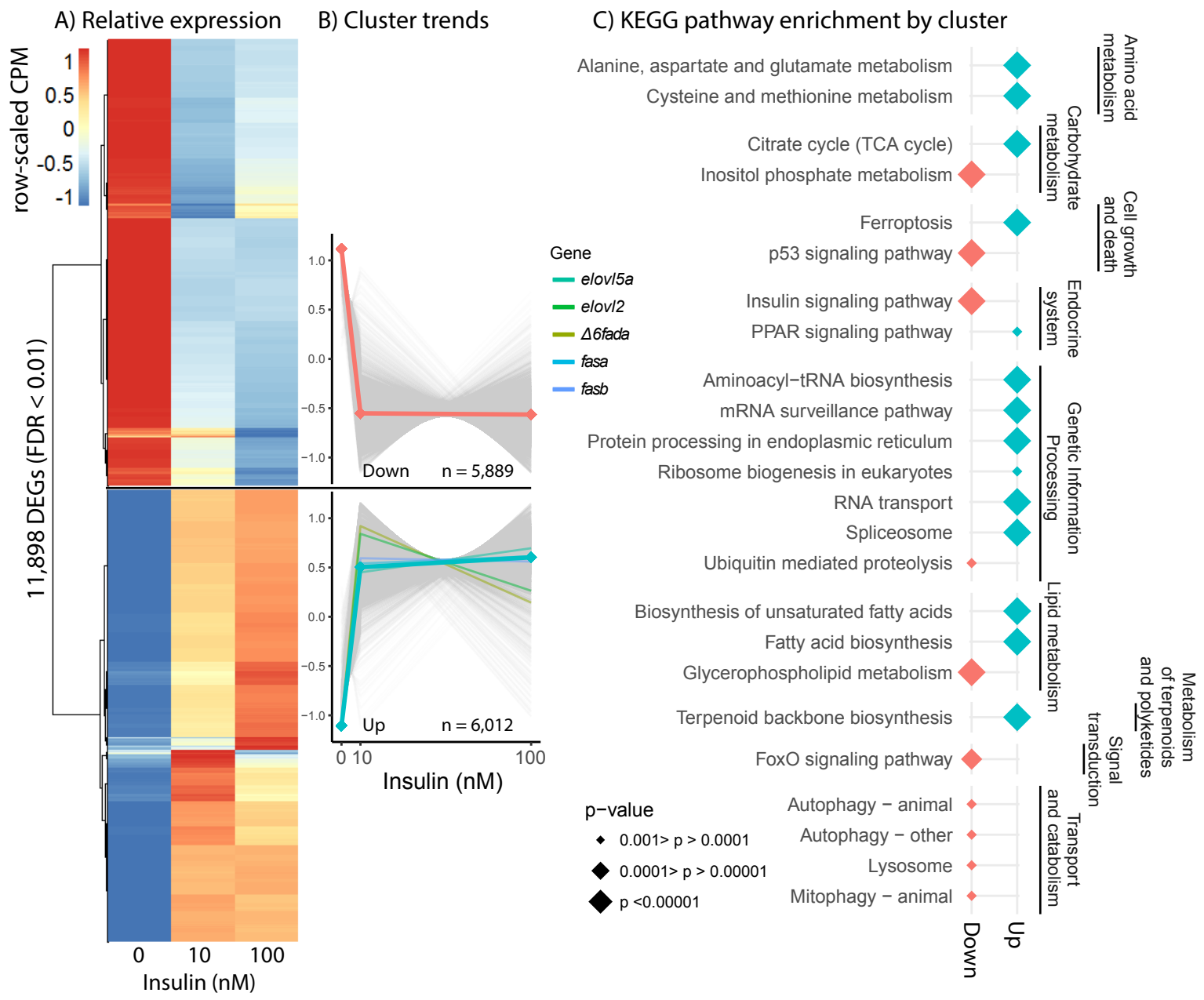

Supplement: Supplemental Information 2 — (A) Heatmap showing changes in relative gene expression with increasing concentration of insulin. Heatmap contains 11,898 genes differentially expressed (FDR < 0.01) at any point in the ALA gradient (ANOVA-like). Transcript abundance is expressed counts per million (CPM) and were row-scaled to highlight changes in individual gene expression. Genes were broken up into two clusters, either downregulated or upregulated. (B) Mean row-scaled CPM (thick lines with points) overlays row-scaled CPM of individual genes (gray). Expression of fatty acid synthase a (fasa), fatty acid synthase b (fasb), elongase 5a (elovl5a), elongase 2 (elovl2), and Δ 6 desaturase a (Δ6fada) are also plotted (thin lines colored by gene). (C) Pathway enrichment analysis on each gene cluster. All pathways shown are significantly enriched (p < 0.001) in the corresponding expression cluster with point size corresponding to p-value. [file peerj-07-7732-s002.pdf]

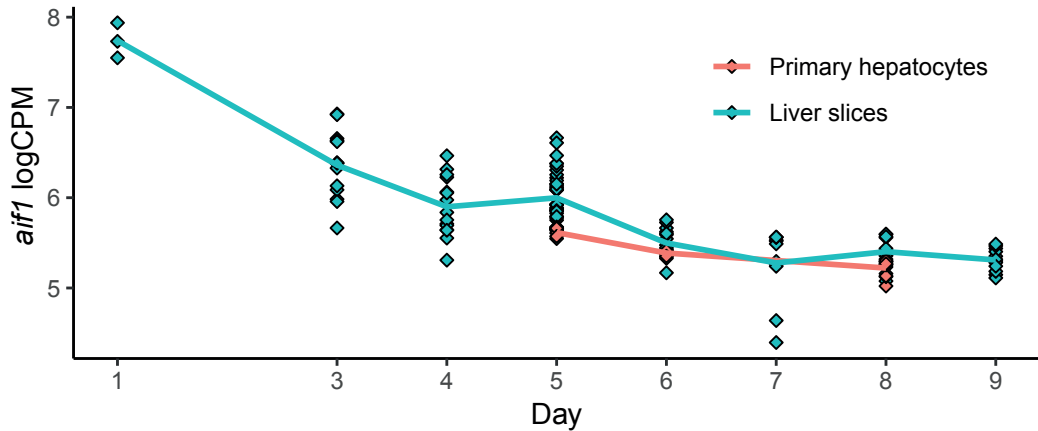

Supplement: Supplemental Information 3 — Gene expression is shown as logCPM. Points represent individual samples and lines represent mean logCPM of those samples. [file peerj-07-7732-s003.pdf]
