# Supplementary material for: Liver slice culture as a model for lipid metabolism in fish
Source: PeerJ. 2019 Sep 17;7:e7732. doi: 10.7717/peerj.7732 (PMC6753922; doi:10.7717/peerj.7732)
Supplement: Supplemental Information 4 — Fatty acid profile of whole liver and liver slices fed increasing amounts of ALA. All values are expressed as triplicate mean percent of total FAs. [file peerj-07-7732-s004.docx]

**Table S1: Fatty acid profile of liver slices incubated with increasing concentration of ALA.**

Fatty acid profile of whole liver and liver slices fed increasing amounts of ALA. All values are expressed as triplicate mean percent of total FAs.

|  |  | ALA concentration (µM) | | | | | |
| --- | --- | --- | --- | --- | --- | --- | --- |
| Fatty acid | Whole liver | 0 | 20 | 40 | 70 | 100 | 140 |
| 14:0 | 1.18 | 1.03 | 0.98 | 1.01 | 1.08 | 1.09 | 0.98 |
| 16:0 | 16.21 | 15.01 | 14.58 | 14.89 | 14.05 | 14.14 | 13.65 |
| 18:0 | 5.8 | 12.29 | 12.08 | 11.66 | 10.40 | 10.33 | 10.56 |
| 20:0 | 0.12 | 0.37 | 0.35 | 0.34 | 0.30 | 0.29 | 0.30 |
| 22:0 | 0.04 | 0.07 | 0.05 | 0.05 | 0.05 | 0.05 | 0.05 |
| Total Saturates | 23.35 | 28.77 | 28.04 | 27.95 | 25.87 | 25.88 | 25.55 |
| 16:1n7 | 1.02 | 0.81 | 0.77 | 0.73 | 0.81 | 0.82 | 0.70 |
| 18:1n9 | 14.11 | 12.84 | 12.81 | 11.40 | 12.79 | 12.29 | 11.17 |
| 20:01 | 1.49 | 2.08 | 2.09 | 1.80 | 1.98 | 1.86 | 1.72 |
| 22:1n9 | 0.08 | 0.22 | 0.26 | 0.20 | 0.23 | 0.21 | 0.20 |
| 24:1n9 | 0.35 | 0.44 | 0.44 | 0.43 | 0.44 | 0.44 | 0.39 |
| Total MUFA | 17.04 | 16.40 | 16.37 | 14.55 | 16.24 | 15.62 | 14.18 |
| 18:2n6 | 4.68 | 3.52 | 3.61 | 3.32 | 3.69 | 3.61 | 3.29 |
| 20:2n6 | 1.05 | 1.43 | 1.49 | 1.41 | 1.37 | 1.33 | 1.33 |
| 20:3n6 | 0.87 | 0.79 | 0.82 | 0.78 | 0.74 | 0.72 | 0.73 |
| 20:4n6 | 3.59 | 2.96 | 3.06 | 3.01 | 2.86 | 2.81 | 2.80 |
| 22:02 | 0.73 | 0.67 | 0.73 | 0.70 | 0.78 | 0.79 | 0.79 |
| Total n-6 PUFA | 10.92 | 9.38 | 9.71 | 9.21 | 9.45 | 9.27 | 8.95 |
| 18:3n3 | 1.17 | 0.88 | 1.44 | 1.88 | 3.04 | 4.12 | 6.52 |
| 20:3n3 | 0.24 | 0.35 | 0.61 | 0.91 | 1.42 | 1.83 | 2.74 |
| 20:5n3 | 6.38 | 3.51 | 3.58 | 3.45 | 3.32 | 3.29 | 3.14 |
| 22:5n3 | 2.07 | 2.26 | 2.34 | 2.33 | 2.14 | 2.12 | 2.09 |
| 22:6n3 | 29.64 | 25.07 | 25.83 | 25.26 | 23.58 | 23.48 | 23.19 |
| Total n-3 PUFA | 39.5 | 32.07 | 33.79 | 33.83 | 33.49 | 34.84 | 37.68 |
