# Supplementary material for: Liver slice culture as a model for lipid metabolism in fish
Source: PeerJ. 2019 Sep 17;7:e7732. doi: 10.7717/peerj.7732 (PMC6753922; doi:10.7717/peerj.7732)
Supplement: Supplemental Information 5 — Quantitative fatty acid profile of whole liver and liver slices fed increasing amounts of ALA. All values are expressed as triplicate mean gram fatty acid per 100 g biomass. [file peerj-07-7732-s005.docx]

**Table S2: Fatty acid profile of liver slices incubated with increasing concentration of ALA.**

Quantitative fatty acid profile of whole liver and liver slices fed increasing amounts of ALA. All values are expressed as triplicate mean gram fatty acid per 100 grams biomass.

|  |  | ALA concentration (µM) | | | | | |
| --- | --- | --- | --- | --- | --- | --- | --- |
| Fatty acid | Whole liver | 0 | 20 | 40 | 70 | 100 | 140 |
| 14:0 | 0.05 | 0.04 | 0.04 | 0.03 | 0.03 | 0.03 | 0.02 |
| 16:0 | 0.62 | 0.55 | 0.54 | 0.39 | 0.36 | 0.39 | 0.29 |
| 18:0 | 0.22 | 0.44 | 0.45 | 0.31 | 0.27 | 0.29 | 0.22 |
| 20:0 | 0.00 | 0.01 | 0.01 | 0.01 | 0.01 | 0.01 | 0.01 |
| 22:0 | 0.00 | 0.00 | 0.00 | 0.00 | 0.00 | 0.00 | 0.00 |
| Total Saturates | 0.90 | 1.05 | 1.05 | 0.74 | 0.67 | 0.72 | 0.54 |
| 16:1n7 | 0.04 | 0.03 | 0.03 | 0.02 | 0.02 | 0.02 | 0.02 |
| 18:1n9 | 0.54 | 0.48 | 0.48 | 0.30 | 0.34 | 0.34 | 0.24 |
| 20:01 | 0.06 | 0.08 | 0.08 | 0.05 | 0.05 | 0.05 | 0.04 |
| 22:1n9 | 0.00 | 0.01 | 0.01 | 0.01 | 0.01 | 0.01 | 0.00 |
| 24:1n9 | 0.01 | 0.02 | 0.02 | 0.01 | 0.01 | 0.01 | 0.01 |
| Total MUFA | 0.65 | 0.62 | 0.61 | 0.38 | 0.43 | 0.44 | 0.30 |
| 18:2n6 | 0.18 | 0.13 | 0.13 | 0.09 | 0.10 | 0.10 | 0.07 |
| 20:2n6 | 0.04 | 0.05 | 0.06 | 0.04 | 0.04 | 0.04 | 0.03 |
| 20:3n6 | 0.03 | 0.03 | 0.03 | 0.02 | 0.02 | 0.02 | 0.02 |
| 20:4n6 | 0.14 | 0.11 | 0.11 | 0.08 | 0.07 | 0.08 | 0.06 |
| 22:02 | 0.03 | 0.02 | 0.03 | 0.02 | 0.02 | 0.02 | 0.02 |
| Total n-6 PUFA | 0.42 | 0.35 | 0.36 | 0.24 | 0.24 | 0.26 | 0.19 |
| 18:3n3 | 0.04 | 0.03 | 0.05 | 0.05 | 0.08 | 0.11 | 0.14 |
| 20:3n3 | 0.01 | 0.01 | 0.02 | 0.02 | 0.04 | 0.05 | 0.06 |
| 20:5n3 | 0.24 | 0.13 | 0.13 | 0.09 | 0.09 | 0.09 | 0.07 |
| 22:5n3 | 0.08 | 0.08 | 0.09 | 0.06 | 0.06 | 0.06 | 0.04 |
| 22:6n3 | 1.14 | 0.91 | 0.96 | 0.67 | 0.60 | 0.65 | 0.49 |
| Total n-3 PUFA | 1.52 | 1.17 | 1.26 | 0.89 | 0.86 | 0.97 | 0.80 |
| Total FA | 3.51 | 3.20 | 3.30 | 2.27 | 2.21 | 2.39 | 1.83 |
